# Prediction and in vitro verification of potential CTL epitopes conserved among PRRSV-2 strains

**DOI:** 10.1007/s00251-017-1004-8

**Published:** 2017-06-07

**Authors:** Simon Welner, Morten Nielsen, Michael Rasmussen, Søren Buus, Gregers Jungersen, Lars Erik Larsen

**Affiliations:** 1 Division for Diagnostics & Scientific Advice - Virology, National Veterinary Institute, Technical University of Denmark, Kemitorvet, Building 204, 2800 Kgs. Lyngby, Denmark; 2 DTU Bioinformatics, Department of Bio and Health Informatics, Technical University of Denmark, Kemitorvet, Building 208, 2800 Kgs. Lyngby, Denmark; 3 Instituto de Investigaciones Biotecnológicas, Universidad Nacional de San Martín, Av. 25 de Mayo y Francia, 1650 San Martin, Cdad. Autónoma de Buenos Aires Argentina; 4 Laboratory of Experimental Immunology, University of Copenhagen, Blegdamsvej 3B, 2200 Copenhagen N, Denmark; 5 Division of Immunology & Vaccinology - Adaptive Immunology, National Veterinary Institute, Technical University of Denmark, Kemitorvet, Building 204, 2800 Kgs. Lyngby, Denmark

**Keywords:** Swine leukocyte antigen, Cytotoxic T lymphocytes, Vaccine, Positional scanning combinatorial peptide library (PSCPL), NetMHCpan, PopCover

## Abstract

Porcine Reproductive and Respiratory Syndrome Virus (PRRSV) is the causative agent of one of the most important porcine diseases with a high impact on animal health, welfare, and production economy. PRRSV exhibits a multitude of immunoevasive strategies that, in combination with a very high mutation rate, has hampered the development of safe and broadly protective vaccines. Aiming at a vaccine inducing an effective cytotoxic T cell response, a bioinformatics approach was taken to identify conserved PRRSV-derived peptides predicted to react broadly with common swine leukocyte antigen (SLA) class I alleles. Briefly, all possible 9- and 10-mer peptides were generated from 104 complete PRRSV type 2 genomes of confirmed high quality, and peptides with high binding affinity to five common SLAs were identified combining the NetMHCpan and positional scanning combinatorial peptide libraries binding predictions. Predicted binders were prioritized according to genomic conservation and SLA coverage using the PopCover algorithm. From this, 53 peptides were acquired for further analysis. Binding affinity and stability of a subset of 101 peptide-SLA combinations were validated in vitro for 4 of the 5 SLAs. Eventually, 23% of the predicted peptide-SLA combinations showed to form complexes with a dissociation half-life ≥30 min. Additionally, combining the two prediction methods proved to be more robust across alleles than either method used alone in terms of predicted-to-observed correlations. In summary, our approach represents a finely tuned epitope prediction pipeline providing a rationally selected ensemble of peptides for future in vivo experiments with pigs expressing the included SLAs.

## Introduction

Porcine Reproductive and Respiratory Syndrome (PRRS) is one of the most important porcine diseases in all swine-producing countries and has a high impact on animal health, welfare, and production economy (Nieuwenhuis et al. 2012; Holtkamp et al. 2013). The causative agent, the PRRS virus (PRRSV), is a small enveloped virus containing a positive-sense single-stranded RNA genome of about 15 kb that encodes 11 open reading frames (ORFs): ORF1a, TF, ORF1b, ORF2a, ORF2b, ORF3, ORF4, ORF5, ORF5a, ORF6, and ORF7 (Fang et al. 2012; Johnson et al. 2011; Meulenberg et al. 1993; Wu et al. 2001). PRRSV exists in two genotypes that were recently classified as distinct species: PRRSV-1 and PRRSV-2. They are both members of the *Arteriviridae* family in the only assigned genus, *Arterivirus*, together with 11 other species including lactate dehydrogenase elevating virus and equine arteritis virus, the latter being type species for the genus. The *Arteriviridae* family is placed together with *Coronaviridae, Roniviridae*, and *Mesoniviridae* in the order *Nidovirales*.

PRRSV infects and replicates within macrophages and eventually kills them. The first cycle of replication occurs in the alveolar macrophages, whereupon the virus can spread to other parts of the body either by means of viremia or inside migrating macrophages. The clinical symptoms appear early after infection, and the most common signs include respiratory symptoms that often leads to fever, lethargy, anorexia, and pneumonia. PRRSV participates as co-factor in polymicrobial syndromes, such as Porcine Respiratory Disease Complex and Porcine Circovirus Associated Disease (Chand et al. 2012). Studies have shown that infectious PRRSV could be isolated from lymphoid tissue more than 150 days after infection even after several months of viral absence in the serum (Wills et al. 1997; Allende et al. 2000). Furthermore, viral replication has been detected for as long as 250 days after infection (Wills et al. 2003). For pregnant gilts and sows infected in late gestation, the virus may infect the endometrium and placenta giving rise to sporadic late-term abortions, early farrowing, and birth of litters mixed with living, stillborn, and mummified fetuses (Zimmerman et al. 1997; Rossow 1998; Karniychuk and Nauwynck 2013). Viremia typically peaks after 10–15 days post infection and in most cases the level of virus in serum is below the detection limit 4 weeks after infection, but the virus may persist in some pigs (Lopez and Osorio 2004). Although the infection is not persistent per se, it is often lifelong since the average lifetime of production pigs is 180 days.

Many attempts have been made to develop an effective vaccine against PRRSV. Various virus attenuation or antigen selection strategies, adjuvants, and delivery systems have been tested including killed virus, modified live virus (MLV), recombinant protein based, and DNA vaccines, as well as their efficacies in terms of viral clearance and relief of symptoms are diverse (reviewed in Renukaradhya et al. 2015a, b). In broad terms, they all succeed to amend the immune response by raising the levels of virus-specific antibodies and/or increasing the cell-mediated immune response (CMI). Commercial MLV vaccines provide moderate to strong protection against a homologous challenge, but none of them seem to be capable of providing cross-protection against heterologous challenges with a sustained protective effect. In addition, the use of MLVs has an immense disadvantage that the attenuated vaccine strain may revert to virulence and start promoting rather than preventing viral infection (Botner et al. 1997; Madsen et al. 1998; Beilage et al. 2009; Jiang et al. 2015). Furthermore, the use of MLV in pregnant animals in the last trimester increases the risk of reproductive failure.

Ideally, a vaccine against PRRSV should induce neutralizing antibodies capable of clearing the virus in its extracellular phase, while a CMI should eliminate infected cells as fast as possible to reduce tissue damage and viral transmission. A key effector cell for this latter task is the cytotoxic T lymphocyte (CTL), having the ability to identify and induce apoptosis of PRRSV-infected cells. Studies have shown that CTLs are indeed present in high numbers at infected locations in the lungs of transplacentally infected animals (Tingstedt and Nielsen 2004) and that the influx of CTLs to the lungs increases during PRRSV infection (Samsom et al. 2000). Although the CTLs are present, their role in clearing the infection is unclear and controversial.

On the skeptical side, Lohse et al. (2004) showed that acute infection appeared to be unaffected by the presence of CTLs since temporary depletion of CTLs during the onset of infection with PRRSV-1 virus neither increased disease nor influenced the ability to clear virus. One study attempted to evaluate the relationship between viral persistence and the presence of CTLs in the blood, the tonsils, the spleen, and the mediastinal lymph nodes in PRRSV-2-infected animals. Although a significant correlation between viral clearance and increased CTL counts in the spleen was observed, a delayed and impaired CMI together with a low level of CTLs was found in the tonsils and lymph nodes, allowing viral persistence in these organs (Lamontagne et al. 2003). In a last example, the cytotoxic activity of peripheral blood mononuclear cells (PBMCs) isolated from Lelystad-infected pigs failed to show PRRSV-specific lysis of infected autologous alveolar macrophages until very late in the experiment. Even following successful expansion of CD3^+^CD8^high^ cells after a 5-day period of restimulation with virus, a PRRSV-specific cytotoxic response was not observed until day 56 post infection, suggesting a PRRSV-induced impairment of the cytotoxic activity (Costers et al. 2009).

On a more optimistic note the CMI against PRRSV-2 was first explored by Bautista (1997), who described a PRRSV-specific lymphocyte proliferation and delayed-type hypersensitivity response, thereby indicating a T cell-specific memory response. Another study argued that a CMI was responsible for the protective immunity of a PRRSV-1 challenge upon vaccination with an MLV vaccine, since a virus-specific interferon-gamma (IFN-γ) response was observed, while no neutralizing antibodies were present (Zuckermann et al. 2007). An in vitro proliferation assay of PBMCs from PRRSV-1 infected cells showed that PBMCs could be expanded upon restimulation with virus and that the cytotoxic activity against K-562 cells increased along with this expansion (López Fuertes et al. 1999).

The observations and conclusions put forward in the literature of CMI responses in relation to PRRSV are thus in many cases contradictory, and it is obvious that more knowledge is needed for a better understanding of the importance of CMI against PRRSV. In this study, a rational approach has been taken to identify potential major histocompatibility complex (MHC) class I restricted epitopes that are conserved among PRRSV-2 strains. Potential epitopes, restricted by five swine leukocyte antigen (SLA) class I alleles, SLA-1*04:01, SLA-1*07:02, SLA-2*04:01, SLA-2*05:02 and SLA-3*04:01, were identified using bioinformatic tools, and subsequently verified in vitro as SLA-binders by affinity and stability assays.

## Materials and methods

### Sequences

#### Verification of genomic data

All available full genome sequences (access date: September 24, 2014) of PRRSV-2 were evaluated and excluded if they failed the criteria of (1) being a wild-type strain, (2) being published, (3) having methionine begin all protein products, and (4) being without non-sense stop codons.

#### Phylogenetic tree

A phylogenetic tree was created in order to illustrate the diversity of the strains used for the prediction. Briefly, for each strain, all naturally occurring protein products (nsp1a, nsp1b, nsp2, nsp2TF, nsp3–6, nsp7a, nsp7b, nsp8–12, ORF2a, ORF2b, ORF3, ORF4, ORF5a, ORF5, ORF6, and ORF7) were concatenated and aligned in CLC (workbench v7.0). The tree was subsequently generated in CLC using the integrated neighbor-joining algorithm with a bootstrap of 1000 replicates.

#### Peptide generation

For each verified strain, all possible 9- and 10-mer peptides were generated in silico from all naturally occurring protein products, excluding peptides spanning post-translational cleavage sites.

#### Swine leukocyte antigen

Five SLA class I alleles were used: SLA-1*04:01, SLA-1*07:02, SLA-2*04:01, SLA-2*05:02 and SLA-3*04:01. Most of these alleles have been shown to be common in Danish pigs (Pedersen et al. 2014) and were readily accessible for in vitro analysis as recombinant biotinylated heavy chains as previously described (Pedersen et al. 2011).

### Epitope bioinformatics

#### NetMHCpan

NetMHCpan (Hoof et al. 2009) version 2.8 was used to predict the binding affinity of the peptides against the five SLA alleles. Version 2.8 has been trained on more than 150,000 quantitative binding data covering more than 150 different MHC-I molecules. The output, being a measure of the binding affinity of a given peptide to a given SLA allele, was converted to a percentile rank score, using SLA-specific standard curves based on the prediction of 200,000 random natural peptides, e.g., a percentile rank score of 2% indicated that the given peptide was among the top 2% best binders to the given SLA out of the 200,000 random natural peptides used for the standard curve.

#### Positional scanning combinatorial peptide library

The positional scanning combinatorial peptide library (PSCPL) method was first described in details by Stryhn et al. (1996) and has since been applied to porcine immunology by Pedersen et al. (2011). Briefly, an SLA-specific scoring matrix providing the average contribution on binding of any amino acid at each position in a 9-mer peptide is used to calculate the overall binding score of a given peptide. The PSCPL experiments providing the scoring matrices for the five SLAs have been performed previously (SLA-1*04:01—Pedersen et al. 2011, SLA-2*04:01—Pedersen et al. 2012, SLA-3*04:01—Pedersen et al. 2014, SLA-1*07:02 and SLA-2*05:02—Lasse Eggers Pedersen, personal communication, April 2014). The matrices for SLA-1*04:01, SLA-2*04:01 and SLA-3*04:01 were based on affinity measurements, while the matrices for SLA-1*07:02 and SLA-2*05:02 were based on stability measurements (shown to give very similar outcomes by Rasmussen et al. (2014)). Since the matrices are based on the binding of 9-mers only, an extrapolation was performed to obtain estimates of 10-mers as described by Lundegaard et al. (2008). The output was converted to a percentile rank score, similar to the above.

#### Combining the methods

Due to the limited amount of porcine MHC-binding data available for training of NetMHCpan, the two methods, NetMHCpan and PSCPL, were combined as this has been shown previously to provide more exact predictions than either method alone (Pandya et al. 2015; Pedersen et al. 2016). A combined rank score was determined for each individual peptide-SLA (pSLA) pair by calculating the harmonic mean of the two method-specific percentile rank scores. Only peptides with a combined rank score ≤2% for at least one of the five SLAs were selected as epitope candidates.

#### Prioritizing the epitope candidates

The PopCover algorithm was used to rank the epitope candidates by iteratively prioritizing the peptides with the broadest SLA allele and strain coverage, while covering the gaps left by previously chosen peptides (Lundegaard and Perez 2010; Buggert et al. 2012).

### In vitro verification of predicted SLA-binders

Based on the bioinformatics described above, 53 peptides (purity >85%, GenScript) were acquired for in vitro verification. Stability and affinity assays were performed on each pSLA with a predicted combined rank score ≤2% using recombinant biotinylated heavy chains of the five SLAs.

#### Stability assays

The stability of the pSLA complexes was determined in vitro using a scintillation proximity assay (SPA) employing the principle of ^125^I-radiolabeled β_2_m dissociation as a measure of pSLA complex stability (Harndahl et al. 2011; Parker et al. 1992b). Briefly, denatured biotinylated recombinant SLA heavy chains were diluted in PBS/0.1% Lutrol F68 to 50 nM and refolded overnight at 18 °C with 2–10 nM ^125^I-radiolabeled β_2_m and ≈50 μM of the peptide to be tested in streptavidin-coated scintillation 384-well FlashPlate PLUS microplates (SMP410A001PK, PerkinElmer). In case of a binding peptide, a scintillation signal was observed and the off-rate was subsequently determined by increasing the temperature to 37 °C and the addition of an excess of unlabeled β_2_m (final concentration 200 nM) while continuously monitoring the scintillation signal in a liquid microplate scintillation plate reader (Topcount NXT, PerkinElmer) for 24 h at 37 °C. The off-rate is equivalent to the peptide-specific dissociation rate and serves as a good measure for pSLA complex stability. The stability values reported are the averages of duplicates in half-life (t½) decimal hours.

#### Affinity assays

Binding affinity of pSLA complexes was determined in vitro using a modified enzyme-linked immunosorbent assay (ELISA) (Sylvester-Hvid et al. 2002; Pedersen et al. 2011). Briefly, 1–2 nM denatured biotinylated recombinant SLA heavy chains were refolded with 15 nM human β_2_m (hβ_2_m) and eight fivefold incremental concentrations of the peptide to be tested spanning from 0 to 13 μM. Following obtained equilibrium after two nights of incubation at RT, the samples were transferred to a streptavidin coated 96-well capture plate (436014, Thermo Scientific) for 1½ h of incubation. Mouse-anti-hβ_2_m, BBM1, and horseradish peroxidase-conjugated goat-anti-mouse IgG (A9917, Sigma-Aldrich) were used as primary and secondary detection antibodies, respectively. Washing steps were performed with 0.05% Tween 20 in PBS. The color reaction of TMB Plus2 (4395A, Kem-En-Tec Diagnostics) was stopped with equivalent amounts of H_2_SO_4_ (0.5 M, cat 30144.294, VWR International), and the plates were read at 450–650 nm using a Multiskan EX ELISA reader (Thermo). OD values were converted to measures of affinity (equilibrium dissociation constant, *K*_D_) using the prefolded biotinylated FLPSDYFPSV/HLA-A*02:01 as standard (Kast et al. 1994), and were again converted to percentile rank scores by the same SLA-specific standard curves that are integrated in NetMHCpan. A minimum of two reliable measurements were aspired for each pSLA combination, and in most cases this was obtained. The results are presented as the range between the minimum and maximum measurements converted to rank scores.

## Results and discussion

### Sequence selection and epitope bioinformatics

**Fig. 1:**
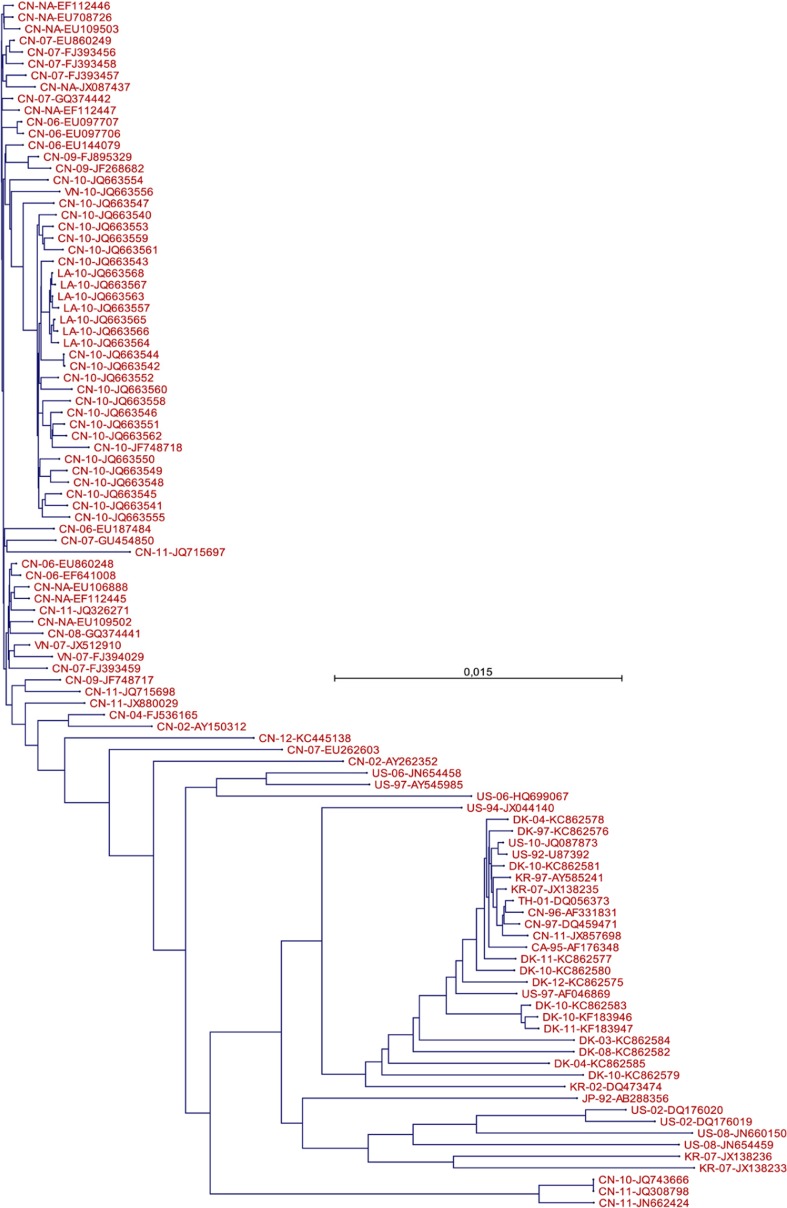
Phylogenetic tree of the 104 strains based on their full proteome. Isolation data (country and year) and accession number are indicated in the legend for each strain, country as a two-letter ISO country code, and year as the last two digits. *NA* no information about isolation year available. *Scale bar* indicates the number of amino acid substitutions per site

Initially, 334 PRRSV-2 full genome (~15.1 kb) sequences were included. Of these, 104 sequences were accepted in accordance with the described verification criteria. Figure 1 illustrates their evolutionary relatedness, while Table 1 shows the year and country of isolation. Out of the 104 accepted strains, 90,939 unique 9- or 10-mer peptides were generated in silico. Binding of each peptide to each of the five SLAs was predicted using the two methods, NetMHCpan and PSCPL. By excluding peptides with a combined rank score >2% with all of the SLAs, the number of unique peptides was reduced to 6140 that were subsequently prioritized using PopCover. Among the top-50 on the PopCover output, four 9-mer peptides nested within top-50 10-mer peptides were excluded, and three peptides further down the list (top-70) were included to give a more even distribution along the genome. In addition, six peptides were included as they have previously been described in the literature in various restimulation studies of PBMCs from pigs immunized with live or attenuated PRRSV-2 virus: Four 17-mers containing the peptides ID43 (TTMPSGFELY), ID50 (NSFLDEAAY), ID53 (MPNYHWWVEH), and ID54 (EVALSAQII), respectively, were found to induce both T cell proliferation and IFN-γ secretion in a screening study of NSP9 and NSP10 (Parida et al. 2012); 15-mers containing peptide ID51 (RGRLLGLLHL) and ID52 (LYRWRSPVI) were found to induce spots in IFN-γ ELISPOT assays when screening GP5 (Vashisht et al. 2008) and the M protein (Wang et al. 2011), respectively. Furthermore, the same ID52 containing 15-mers as above was included in a peptide-based vaccine with the N-terminal part of the heat chock protein Gp96 as adjuvant. Restimulation with this peptide of PBMCs from the immunized animals was shown to induce lymphocyte proliferation with a Th1-type cytokine bias, and the immunized piglets exhibited milder clinical symptoms, lower viremia, and less pathogenic lesions than non-immunized piglets upon challenge with a highly pathogenic PRRSV strain (Chen et al. 2013).

**Table 1:** Distribution of the 104 strains according to isolation year and country

Country of isolation		NA	1992	1994	1995	1996	1997	2001	2002	2003	2004	2006	2007	2008	2009	2010	2011	2012	Percent
Canada	CA				1														1
China	CN	8				1	1		2		1	6	8	1	3	23	7	1	60
Denmark	DK						1			1	2			1		5	2	1	13
Japan	JP		1																1
Laos	LA															7			6.7
South Korea	KR						1		1				3						4.8
Thailand	TH							1											1
USA	US		1	1		1	2		2			1		2		1			11
Vietnam	VN												2			1			2.9
	%	7.7	1.8	1	1	1.9	4.8	1	4.8	1	2.9	6.7	13	3.8	2.9	36	8.7	1.9	100

*NA* not available

Unfortunately, none of these studies had a clear phenotypic description of the responding cells, nor had their test animals been SLA genotyped.

In total, 54 peptides were ordered from Genscript, but only 53 were received as peptide ID14 could not be synthesized (Table 2).

**Table 2:** Overview of the results obtained from the in vitro studies

ID	Sequense	Locus	Cons. 104 strains (%)	SLA-1*04:01					SLA-1*07:02					SLA-2*04:01					SLA-2*05:02				
				Stab. (h)	Aff. (nM)	PSCPL (rank)	NMP (rank)	Comb. (rank)	Stab. (h)	Aff. (nM)	PSCPL (rank)	NMP (rank)	Comb. (rank)	Stab. (h)	Aff. (nM)	PSCPL (rank)	NMP (rank)	Comb. (rank)	Stab. (h)	Aff. (nM)	PSCPL (rank)	NMP (rank)	Comb. (rank)
1	RTILGTNNF	nsp9	98	0.1	1030^a^	3	1.5	2			32	6	10.1	0.1	–	1.5	1.5	1.5			50	4	7.4
2	YAQHMVLSY	nsp9	100	–	–	32	0.4	0.8	0.9	4	1.5	0.03	0.1	1.1	60	15	1	1.9			50	4	7.4
3	RSTPAIVRWF	nsp9	97	0.1	–	0.25	3	0.5			50	32	39	–	–	0.8	4	1.3			50	10	16.7
4	YSFPGPPFF	nsp9	71	0.2	37^a^	0.8	0.5	0.6	0.2	18,708	8	1	1.8	–	–	0.5	1.5	0.8	0.1	–	50	0.17	0.3
5	RALPFTLSNY	ORF2a	96			9	1.5	2.6	0.3	12	2	1.5	1.7	0.1	–	1.5	1.5	1.5			50	15	23.1
6	KSGHPHGLLF	nsp9	95	0.1	–	50	1	2			50	7	12.3			7	3	4.2			50	32	39
7	QVYERGCRWY	nsp1a	100	0.3	682	2	2	2	4.5	168	5	0.8	1.4	0.7	209^a^	4	0.17	0.3			50	32	39
8	GGNWFHLEW	ORF3	99			10	5	6.7			50	4	7.4	0.2	16,627	1.5	3	2			50	15	23.1
9	IVYSDDLVLY	nsp9	99	–	–	6	0.8	1.4	0.5	13	0.5	0.1	0.2	–	–	1.5	1	1.2			50	9	15.3
10	KVAHNLGFYF	nsp11	95	0.3	122	15	0.4	0.8			2	4	2.7			10	4	5.7			50	4	7.4
11	TRARHAIFVY	nsp10	95			50	15	23.1	0.3	60	0.25	1.5	0.4	0.2	–	7	0.8	1.4			50	50	50
12	LSFSYTAQF	ORF3	99			5	3	3.8			32	1.5	2.9	1.3	73	3	0.3	0.5	–	–	50	0.8	1.6
13	FTWYQLASY	nsp12	97	0.1	92^a^	8	0.8	1.5	0.2	62	4	0.15	0.3	9.1	2	0.025	0.25	0.0	–	–	50	7	12.3
15	TLSNYRRSY	ORF2a	97	–	–	1.5	1.5	1.5	0.3	29,504	1	1.5	1.2	–	–	15	1	1.9			32	50	39
16	SGHPHGLLF	nsp9	96			10	4	5.7			9	4	5.5	–	–	15	0.8	1.5			50	15	23.1
17	RTAIGTPVY	ORF4	96	0.5	57	1	0.25	0.4	0.2	1852	4	1	1.6	–	385^a^	1	0.8	0.9			50	10	16.7
18	YTAQFHPEIF	ORF3	98	–	–	3	1	1.5			9	2	3.3	0.4	–	0.1	2	0.2	0.2	555^a^	32	0.5	1
19	LSDSGRISY	ORF7	95	1.1	10	0.8	0.112	0.2	0.2	393	15	0.3	0.6	0.2	4182^a^	1	1.5	1.2			50	15	23.1
20	ASYVWVPLTW	nsp3	88			4	3	3.4			50	4	7.4	0.1	17,142	2	0.5	0.8			50	3	5.7
21	KVAHNLGFY	nsp11	95	1.5	4	50	0.2	0.4	2.8	11	0.5	1.5	0.8	–	–	6	0.5	0.9			50	16	24.2
22	KIFRFGSHKW	NSP1b	88			5	2	2.9			50	4	7.4	0.1	9^a^	15	1	1.9			50	6	10.7
23	NISAVFQTYY	ORF3	93	0.1	413	2	1.5	1.7	0.9	6	50	0.3	0.6	–	862	1.5	2	1.7			50	15	23.1
24	RTAPNEIAF	nsp2	66	2.1	4	0.125	0.4	0.2			9	6	7.2	0.1	–	0.8	4	1.3			32	4	7.1
25	ASDWFAPRY	ORF2a	92	4.9	2	0.8	0.05	0.1	0.2	71	9	0.17	0.3	–	–	3	0.8	1.3			50	32	39
26	RMMGHAWTPL	nsp5	99			5	2	2.9			32	15	20.4			50	4	7.4			15	3	5
27	RPFFSSWLV	ORF3	100			50	50	50	37.4	1	0.15	5	0.3			50	32	39			32	3	5.5
28	FVLSWLTPW	nsp5	90	–	–	3	1.5	2			32	2	3.8	13.7	3	3	0.8	1.3	–	–	50	0.4	0.8
29	MVNTTRVTY	nsp10	91	0.1	206^a^	3	0.8	1.3	0.2	47	1.5	0.17	0.3	–	–	4	1	1.6			32	9	14
30	CVFFLLWRM	nsp5	100			50	15	23.1	0.2	283	0.8	5	1.4			50	8	13.8			15	7	9.5
31	ATQEWLSRMW	nsp2	85	–	–	3	0.8	1.3			50	6	10.7	0.1	50,000^a^	5	1	1.7			50	2	3.8
32	VAHNLGFYF	nsp11	95			16	4	6.4			3	3	3			50	4	7.4			50	2	3.8
33	ITANVTDENY	ORF4	89	0.1	–	5	0.8	1.4	0.3	69	7	0.8	1.4	–	–	0.4	0.8	0.5			50	6	10.7
34	SSEGHLTSVY	ORF3	88	–	–	0.25	0.12	0.2	0.2	12,701^a^	32	0.8	1.6	0.1	1692^a^	2	0.05	0.1			50	15	23.1
35	RTILGTNNFI	nsp9	94			15	4	6.3			50	32	39			15	4	6.3	0.3	60	5	0.4	0.7
36	LTAALNRNRW	nsp5	85	–	–	2	1.5	1.7			50	3	5.7	3.6	40	0.8	1	0.9	–	–	50	0.5	1
37	RTMLFTPSQL	nsp3	93			15	1.5	2.7			50	15	23.1			6	4	4.8	–	–	32	0.8	1.6
38	LSASSQTEY	nsp2	79	0.1	91^a^	3	0.5	0.9	0.2	479	50	0.5	1	0.2	–	0.5	1	0.7			50	6	10.7
39	VRWFAANLLY	nsp9	93			50	16	24.2	2.7	44	0.8	1	0.9			7	3	4.2			50	50	50
40	RMTSGNLNF	nsp1a	100	–	–	0.25	0.8	0.4			15	4	6.3			9	8	8.5			50	7	12.3
41	AGACWLSAIF	nsp1a	99	–	–	0.8	6	1.4			32	15	20.4			7	3	4.2			50	15	23.1
42	SAIPFRAPFF	nsp2	68			8	3	44			4	3	3.4	–	–	3	0.8	1.3			32	1.5	2.9
43	TTMPSGFELY	nsp9	75	–	576	0.8	0.25	0.4	0.8	6	0.5	0.05	0.1	0.2	1838^a^	0.4	0.2	0.3	–	–	50	0.25	0.5
44	MSWRYSCTRY	ORF5	74	–	–	7	0.8	1.4	0.5	87	8	0.1	0.2	1.5	15	0.8	0.05	0.1			50	4	7.4
45	SSAFFLRYF	nsp5	66			50	2	3.8			15	5	7.5	0.1	–	0.8	0.5	0.6			50	4	7.4
46	ALATAPDGTY	nsp3	100	–	607^a^	50	0.5	1	0.1	2736	0.8	1.5	1			7	3	4.2			50	32	39
47	RVRMGVFGCW	nsp2	99			32	7	11.5			50	16	24.2	–	–	1.5	3	2			50	32	39
48	WGVYSAIETW	ORF6	97			32	32	32			50	10	16.7	0.2	21,433	0.5	5	0.9			50	7	12.3
49	FLNCAFTFGY	ORF6	97	–	–	4	0.8	1.3	0.3	20	1	0.25	0.4			2	3	2.4			50	15	23.1
50	NSFLDEAAY	nsp10	96			2	3	2.4	0.1	43	9	0.5	0.9	–	–	5	0.4	0.7			50	15	23.1
51	RGRLLGLLHL	ORF6	100			32	50	39			50	50	50			32	15	20.4			50	50	50
52	LYRWRSPVI	ORF5	94			50	50	50			50	50	50			50	32	39			15	50	23.1
53	MPNYHWWVEH	nsp9	100			50	50	50	0.6	32	0.5	3	0.9			10	32	15.2			50	32	39
54	EVALSAQII	nsp9	89			15	32	20.4			32	32	32			15	15	15	18.3	2	0.5	1.5	0.8

Left section lists the individual peptides represented by ID, sequence, locus of origin, and percent conservation among the 104 ancestry strains. Right section presents the measured and predicted values for the respective peptide-SLA combinations. Only combination predicted to have a combined rank score ≤2% were measured. From left to right, the columns represent measured stability (average dissociation half-life in decimal hour (h)), measured affinity (average equilibrium dissociation constant (nM)), predicted binding by PSCPL (rank), predicted binding by NetMHCpan (rank), and combined predicted binding by calculating the harmonic mean of PSCPL and NetMHCpan (rank)

En dash (−), no successful measurement (stability or affinity) could be obtained; *empty field*, not tested due to a predicted combined rank score >2% for the given peptide-SLA combination

^a^Only one successful affinity measurement could be obtained

Due to internal errors, the NetMHCpan prediction was performed on SLA-2*05:01 instead of the correct SLA-2*05:02. Even though the two alleles are genetically very similar, their peptide binding specificities are only partly overlapping. Unfortunately, the mistake was not discovered before the peptides were purchased and as a consequence, only 9 out of the 53 peptides were predicted as binders to SLA-2*05:02 while this number was formerly believed to be 24. For obvious reasons, this insight would have resulted in a different PopCover output and hence a different choice of peptides for purchase.

### Experimental verification of predicted pSLA complexes

For each of the 53 peptides, each pSLA combination that was predicted to have a combined rank score ≤2% was tested in vitro for their individual binding characteristics in terms of affinity and stability. The results are presented in Table 2. Note that only SLA-1*04:01, SLA-1*07:02, SLA-2*04:01, and SLA-2*05:02 were included in this experimental validation, as no functional assay could be generated for SLA-3*04:01.

While the affinity represents the strength of a peptide-MHC (pMHC) interaction, the stability represents the longevity of this interaction once established. The two properties are mechanistically interrelated but are not mutually redundant, meaning that a peptide having a strong affinity will not necessarily form a highly stable complex, and vice versa. Obviously, the probability of a pMHC complex on the surface of a given cell to encounter and be recognized by an extremely rare circulating CTL with a cognate receptor is proportionate to how long this peptide is being displayed on the cell surface—the stability. Likewise, this probability is also proportionate to the number of successfully formed pMHC molecules in the first place—the affinity. Factors other than affinity and stability may also play a role, such as the level of protein being expressed in the cytosol from which the peptide is derived, and the rate at which the MHC molecule is internalized after peptide presentation on the cell surface.

In the earliest works of characterizing the pMHC interaction, both affinity and stability was given considerable focus (Buus et al. 1987; Parker et al. 1992a, b). Regardless, the vast majority of available pMHC binding data is in the form of affinity data since the acquisition of stability data has previously been cumbersome and laborious. Recently, the SPA method used in this study, being a high-throughput one-step method for measuring pMHC stability was developed by Harndahl et al. (2011), and shortly after, Harndahl et al. (2012) showed that immunogenic peptides tend to be more stably bound to MHC-I molecules than non-immunogenic peptides, suggesting to focus on stability rather than affinity as a determinant for peptide immunogenicity. In the wake of this, the NetMHCstab and NetMHCstabpan servers were recently established (Jørgensen et al. 2014; Rasmussen et al. 2016). Unfortunately, these servers have so far only been trained with human data, and could therefore not be implemented in this study.

In the light of the indicated proportionality between immunogenicity and stability, it has become convenient to define a threshold separating binders from non-binders. While the NetMHCstab server defines the thresholds for weakly and strongly stable pMHC complexes to be a t½ ≥ 2 h and t½ ≥ 6 h, respectively, other studies have been less strict and included pMHCs with t½ ≥ 30 min. Out of the 101 pMHC complexes tested in this study, 23 of these exhibited a t½ ≥ 30 min (5/30 pSLA-1*04:01, 10/26 pSLA-1*07:02, 7/36 pSLA-2*04:01, and 1/9 pSLA-2*05:02). Ten of these had a t½ ≥ 2 h, and four had a t½ ≥ 6 h. Interestingly, peptide ID54 (EVALSAQII), which was included due to its previous mention in the literature, was measured to bind very stably to SLA-2*05:02 (t½ = 18.3 h), hinting that the responsive animals used by Parida et al. (2012) could have expressed this particular allele.

### Correlations between predicted and measured values

**Fig. 2:**
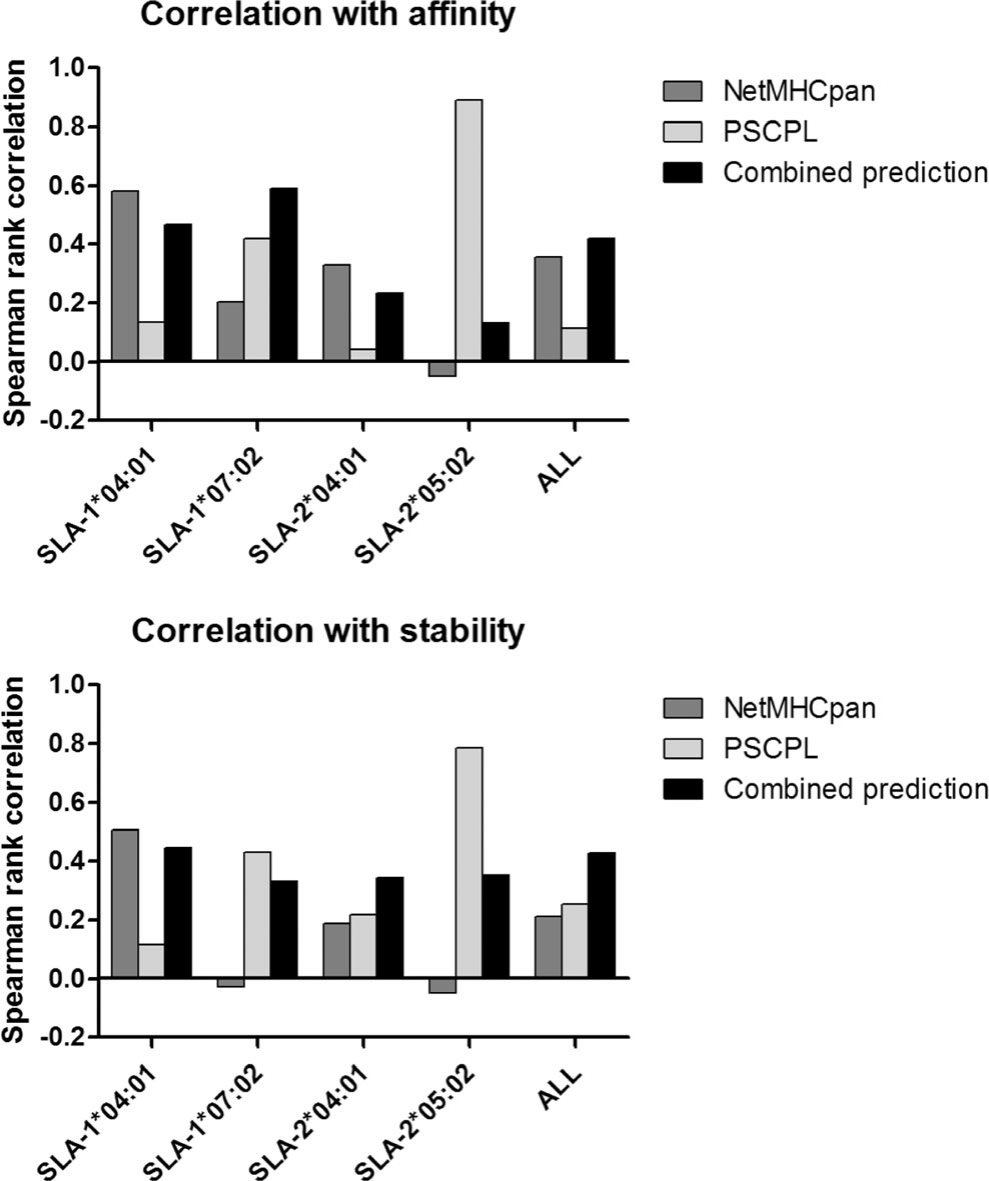
Correlation analysis between measured binding affinity/stability and predicted rank values for the three methods NetMHCpan, PSCPL, and combined prediction. Correlations were quantified in terms of Spearman rank correlation. *ALL* gives the correlation values for the combined data set of the four SLA molecules

To quantify the performance of the three prediction strategies employed for peptide selection, a correlation analysis between the predicted rank score and the measured binding affinity and binding stability values was performed. The analysis was limited to the molecules SLA-1*04:01, SLA-1*07:02, SLA-2*04:01, and SLA-2*05:02, and the results are displayed in Fig. 2. From this analysis, it is apparent that none of the two methods, NetMHCpan and PSCPL, consistently outperformed the other. The PSCPL method achieved the highest performance of the two methods for 50% of the SLA alleles on the binding affinity data and for 75% of the alleles in the stability data. Each method performed very poorly with close to zero or negative correlations in at least one case each. In contrast to this, the performance of the combined method was consistently high across all four SLA alleles, thus achieving the highest performance of the three methods on both the affinity and stability data when evaluated on the data set combined of all SLA measurements. This finding thereby confirmed the earlier finding that combining prediction of NetMHCpan and PSCPL leads to a superior performance for identifying peptide binders to MHC molecules characterized by limited or no binding data (Rasmussen et al. 2014; Hansen et al. 2014).

**Fig. 3:**
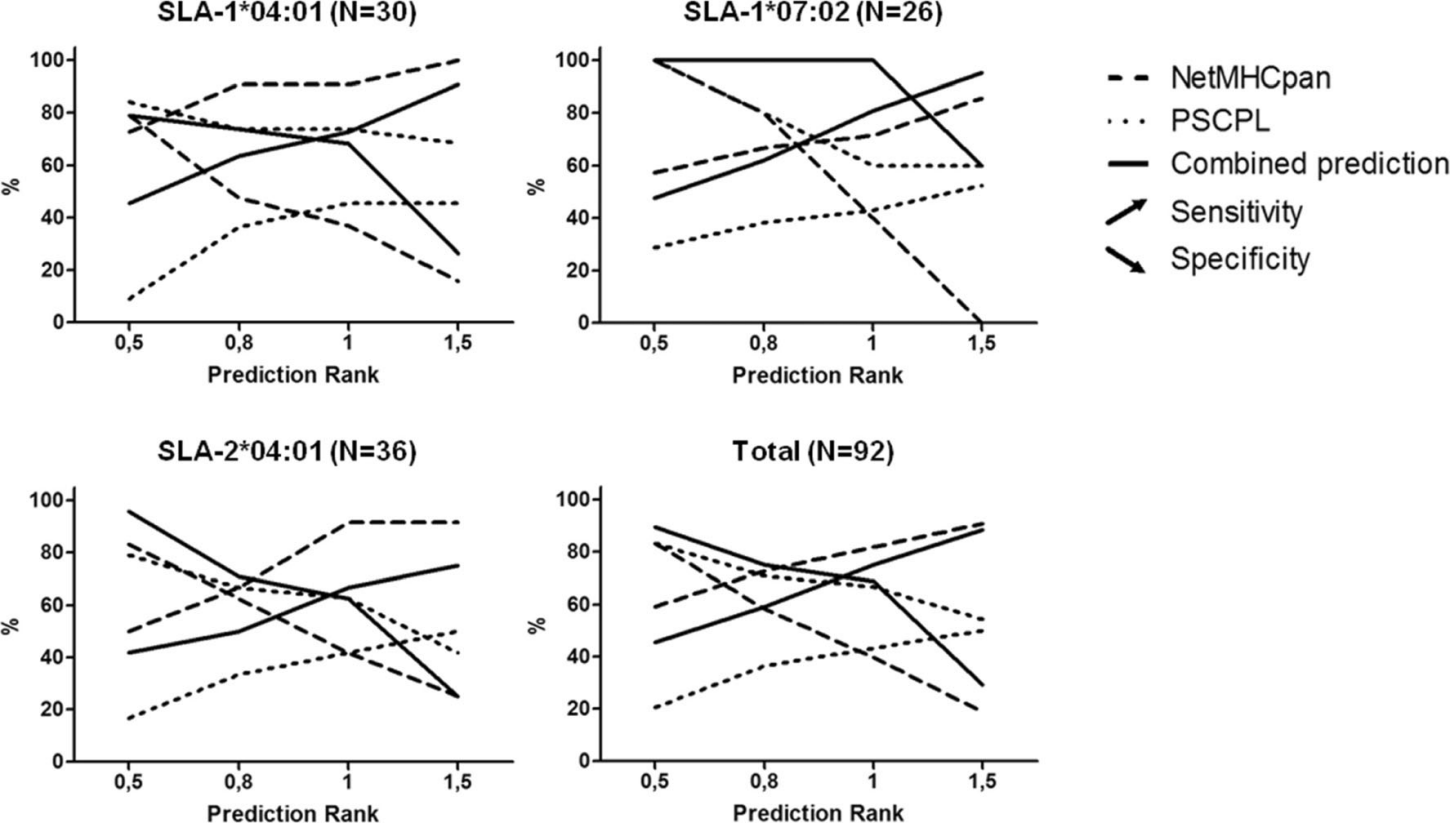
Analysis of sensitivity and specificity of the three methods (NetMHCpan, PSCPL and combined prediction) with relation to the three alleles (SLA-1*04:01, SLA-1*07:02, and SLA-2*04:01). Values of sensitivity and specificity were calculated based on four different values of predicted rank: 0.5, 0.8, 1 and 1.5 (observed binders and non-binders were classified as described in the text). Sensitivity indicates the percentage of observed binders identified below or equal to the four predicted rank values. Specificity indicates the percentage of observed non-binders identified above the four predicted rank value

We next extended this analysis to include an evaluation of the sensitivity (true positive rate) and specificity (true negative rate) of the respective methods. Due to the inconsistencies between the SLA allele used to selected peptides and the allele actually used in the study for the SLA-2*05:01/:02, the SLA-2*05:01 allele was excluded from this analysis, which was hence limited to SLA-1*04:01, SLA-1*07:02, and SLA-2*04:01. The results are given in Fig. 3, depicting the sensitivity and specificity as a function of the prediction rank threshold for the three respective methods for the three different SLA molecules. Due to the fact that different MHC molecules display very different binding potential when it comes to both affinity (Paul et al. 2013; Nielsen and Andreatta 2016) and stability (Rasmussen et al. 2016), an allele-specific affinity threshold was defined to distinguish between observed binders and non-binders. This threshold was defined from the 1% percentile affinity score obtained by predicting binding to 200,000 random natural peptides using NetMHCpan (version 2.9). We are aware that using this approach might introduce a bias in favor of the NetMHCpan prediction. Nevertheless, we regarded this as a better estimate and representative of the individual alleles than the hitherto general definition of a uniform threshold at 500 nM that does not account for any allele-specific variation (Yewdell and Bennink 1999). As expected, the obtained allele-specific affinity thresholds demonstrated substantial variations with values spanning from 546 nM (SLA-1*04:01) over 1193 nM (SLA-1*07:02) to 3415 nM (SLA-2*04:01).

In general, a high-performance method should have a point on the graphs with high sensitivity and specificity values. Given this, the value corresponding to the cross-point of the sensitivity and specificity curves can be taken as a measure of predictive performance of a given method. Using this performance measure, NetMHCpan demonstrates a general very high performance, with cross-point values for the three SLA molecules in the range 0.64–0.75, meaning that on average 70% of the binding peptides are captured at a false positive rate of 30% (Fig. 3). For the PSCPL method, these values are substantially lower and in two of the three cases, no cross-point is identified in the rank score range included in the analysis, suggesting a low sensitivity of this approach. However, even in this situation, the combination of the two approaches leads to an overall improved performance, with a substantially improved cross-point (0.86 compared to 0.68) value for SLA-1*07:02. These findings thus consolidate the earlier conclusion that integrating PSCPL and NetMHCpan predictions leads to overall superior performance compared to any of the individual methods alone.

### Perspectives of an epitope based vaccine strategy

The central concept of vaccinology is defined by the proper presentation of antigen to the immune system. For a vaccine to induce a CMI, more specifically, the antigen is presented on a MHC-I molecule as an 8–10-mer epitope for subsequent recognition by a cognate cytotoxic T cell. Applying this to a real-life vaccine trial, this concept splits up into three cardinal points that should be considered during the development of an epitope-based vaccine: (1) *Pathogen diversity*. While it would be very unlikely to identify a single immunogenic epitope expressed by all circulating strains of the target pathogen, the epitopes included in the vaccine should reflect the diversity of the circulating target pathogen. Choosing conserved epitopes must be regarded as the only rational approach, as this would not only ensure the highest degree of pathogen coverage attained by the lowest number of epitopes but would also exclude epitopes that are dispensable for the pathogen. It is likely, however, that a higher selection pressure on conserved epitopes may lead to the employment of mechanisms to prevent these epitopes from being immunogenic. Such mechanisms could result in a level of surface display sufficiently low to avoid CTL priming and activation. If this is the case, one could speculate that an artificially raised level of display in terms of a vaccine could activate cognate CTLs to such an extent that they would recognize and kill cells displaying an otherwise negligible level of epitopes, i.e., cells infected with wild-type virus. (2) *Herd diversity*. Currently, 216 SLA class I alleles are known, including 62 SLA-1, 61 SLA-2, and 32 SLA-3 alleles. The majority of the SLA alleles are published in the Immune Polymorphism Database (http://www.ebi.ac.uk/ipd/mhc/sla/). This number is relatively small compared to the known human MHC-I diversity counting several thousand proteins and is likely to be a consequence of scientific focus and limited genetic diversity within the swine industry. Although their peptide specificities overlap to some extent, the limited number of epitopes included in a vaccine should be selected to match the allelic diversity of a target population. (3) *Epitope immunogenicity*. While the notion of being immunogenic is not synonymous with providing protection, it is definitely a prerequisite. Beyond epitope abundance, the underlying mechanisms of epitope immunogenicity involve six steps: (i) cleavage of a cytosolic protein into smaller fragments by the immuno- or conventional proteasome; (ii) transportation of these fragments into the endoplasmic reticulum by TAP; (iii) N-terminal trimming of the fragments by aminopeptidases (Serwold et al. 2002); (iv) association of the peptide to the MHC-I molecule; (v) vesicular transportation of the pMHC complex to the cell surface; and (vi) recognition of the pMHC by a CTL with a cognate T cell receptor. The steps i-iii relate to the preprocessing of the peptides, and even though information can be gained from the specificities of the involved enzymes and transporters, this information has no impact on the NetMHCpan predictions used in this study (Peters et al. 2003; Nielsen et al. 2005). Consequently, it was decided not to take this into account. The steps iv and vi, however, represent the most selective steps in the MHC-I presentation pathway and recognition by circulating T cells, respectively.

In this study, we have defined and characterized an ensemble of potential CTL epitopes conserved among PRRSV-2 strains for the future development of an epitope-based vaccine. The abovementioned three cardinal points have been met by (1) deriving all 9–10-mer peptides from a database of 104 wild-type strains; (2) designing an ensemble of 53 epitope candidates predicted for an optimal representation of antigen to a fictive target population expressing the five SLAs in question. This was done by the use of bioinformatic tools for epitope prediction (NetMHCpan and PSCPL) and subsequent ranking of epitope candidates (PopCover); and 3) verification of MHC binding of the 53 selected epitope candidates to the five SLAs using in vitro pMHC stability and affinity assays. In addition to this, the correlation between predicted and observed binding data was analyzed for NetMHCpan, PSCPL, and the combination of the two. In accordance with earlier studies, none of the individual methods consistently outperformed the other, and the combination of the two performed a robust prediction across all SLAs with a relatively high correlation.

## Concluding remarks

In order to obtain an ensemble of epitopes that can provide protection to a population of diverse haplotypes, the ensemble must consist of epitopes that collectively will bind to the majority of haplotypes present in the population. The PopCover algorithm was employed in this study to prioritize between the peptides based on both their degree of conservation and their “promiscuity” with regard to SLA binding. While these two factors are central in the definition of a peptide ensemble, weight coefficients could be adjusted in relation to the individual strains, peptides, and SLA alleles, in order to fine-tune the definition of the ensemble. Weight coefficients related to the individual strains should be set to compensate for a bias induced by an overrepresentation of similar strains in cases where this would reflect an intensely sequenced incidence of disease rather than reflecting the actual diversity of the strains. As an example, this study includes seven viruses isolated in Laos in 2010. As seen in Fig. 1, these strains are very closely related and do most likely represent seven variants of the same strain rather than seven different strains. Consequently, the weight coefficients of these should be adjusted to make them have a collective impact corresponding to a single strain. For the individual peptides, weight coefficients should be given to reflect their relative levels of expression. In case of PRRSV, the expression levels differ substantially between loci, which are strongly influenced by the programmed ribosomal frameshifting sites. According to Fang et al. (2012), only about 15% of the translation initiated at the beginning of ORF1a will proceed to ORF1b. As a result, peptides derived from ORF1b will be much less abundant compared to peptides derived from ORF1a, and should be reflected accordingly by their weight coefficients. Also, for the MHC alleles, a weight coefficient could be implemented to reflect their relative levels of expression. On that node, we have observed that the average level of complementary DNA (cDNA) derived from SLA-3 mRNA was less than 10% of the overall SLA cDNA. The remaining 90% were more or less evenly distributed between SLA-1 and SLA-2 derived cDNA (unpublished data). This may, however, stand in contrast to the abundance of a given MHC allele in the herd in general, which in case of SLA-3 was indeed found to be quite abundant in some populations (Pedersen et al. 2014). Thus, two weight coefficients could be given for the MHCs, reflecting both the relative levels of expression in the individuals and the levels of abundance in the population.

It is obvious that the definition of an optimal epitope ensemble for the induction of an immune response against a pathogen on the population level is not straightforward. In the current study, none of the abovementioned weight coefficients have been used to balance the epitope candidates. Because of this, and because of confusion regarding SLA-2*05:01 and SLA2*05:02, the presented ensemble of 53 peptides is most probably different from how it would be composed otherwise. Nonetheless, 53 conserved peptides have been analyzed in vitro for their binding capacities to five different SLAs. The biological significance of these results are yet to be tested, and may ultimately aid in the development of a CTL-activating vaccine against PRRSV.
